# Supplementary material for: Mitochondrial phenotypes in purified human immune cell subtypes and cell mixtures
Source: eLife. 2021 Oct 26;10:e70899. doi: 10.7554/eLife.70899 (PMC8612706; doi:10.7554/eLife.70899)
Supplement: Transparent reporting form [file elife-70899-transrepform1.docx]

***eLife’s* transparent reporting form**

We encourage authors to provide detailed information *within their submission* to facilitate the interpretation and replication of experiments. Authors can upload supporting documentation to indicate the use of appropriate reporting guidelines for health-related research (see [EQUATOR Network](http://www.equator-network.org/%20)), life science research (see the [BioSharing Information Resource](https://biosharing.org/)), or the [ARRIVE guidelines](http://www.plosbiology.org/article/info:doi/10.1371/journal.pbio.1000412) for reporting work involving animal research. Where applicable, authors should refer to any relevant reporting standards documents in this form.

If you have any questions, please consult our Journal Policies and/or contact us: [editorial@elifesciences.org](mailto:editorial@elifesciences.org).

**Sample-size estimation**

- You should state whether an appropriate sample size was computed when the study was being designed
- You should state the statistical method of sample size computation and any required assumptions
- If no explicit power analysis was used, you should describe how you decided what sample (replicate) size (number) to use

Please outline where this information can be found within the submission (e.g., sections or figure legends), or explain why this information doesn’t apply to your submission:

A total of 340 samples were collected from 22 individuals of different ages, ethnicities, and gender, recruited from the United States and is explained in the “Methods” section (page 32 and 35).

For cell subtype comparisons, the main outcome of this study, the sample size is n=12-16 individuals (6 most abundant cell subtypes from 21 individuals), which is sufficient to detect small to medium effect size differences. The observed effect sizes between cell subtypes are large to extremely large (Hedges' g<2). For correlations with age, sex, and biomarkers, this sample size is sufficient to detect large effect sizes only.

For the repeat participant study, the sample size is n=9 repeated observations, which is also sufficient to detect small to moderate effect sizes. Again, the cell type differences are the same as in the cohort. The number of observations, which were in part limited by feasibility of consecutive repeated-measures in the same participant, is sufficient to generate robust estimates of variation over time. For correlations with biomarkers, this sample size is sufficient to detect large effect sizes only.

**Replicates**

- You should report how often each experiment was performed
- You should include a definition of biological versus technical replication
- The data obtained should be provided and sufficient information should be provided to indicate the number of independent biological and/or technical replicates
- If you encountered any outliers, you should describe how these were handled
- Criteria for exclusion/inclusion of data should be clearly stated
- High-throughput sequence data should be uploaded before submission, with a private link for reviewers provided (these are available from both GEO and ArrayExpress)

Please outline where this information can be found within the submission (e.g., sections or figure legends), or explain why this information doesn’t apply to your submission:

Mitochondrial assays were ran in triplicates. The information on the number of replicates used for analyses are detailed in Supplementary file 3, “Methods” section (pages 33), and Appendix 1 (page 4).

No data was excluded.

**Statistical reporting**

- Statistical analysis methods should be described and justified
- Raw data should be presented in figures whenever informative to do so (typically when N per group is less than 10)
- For each experiment, you should identify the statistical tests used, exact values of N, definitions of center, methods of multiple test correction, and dispersion and precision measures (e.g., mean, median, SD, SEM, confidence intervals; and, for the major substantive results, a measure of effect size (e.g., Pearson's r, Cohen's d)
- Report exact p-values wherever possible alongside the summary statistics and 95% confidence intervals. These should be reported for all key questions and not only when the p-value is less than 0.05.

Please outline where this information can be found within the submission (e.g., sections or figure legends), or explain why this information doesn’t apply to your submission:

Each Figure legend contains the statistical information and details of tests concerning the data represented in the Figure. P-values and effect sizes can also be found in the “Results” section (pages 5-26). A summary of all statistical analysis performed, along with other statistical information can be found in the “Methods” section (page 35) and in the “Appendix 1” (page 8).

(For large datasets, or papers with a very large number of statistical tests, you may upload a single table file with tests, Ns, etc., with reference to sections in the manuscript.)

**Group allocation**

- Indicate how samples were allocated into experimental groups (in the case of clinical studies, please specify allocation to treatment method); if randomization was used, please also state if restricted randomization was applied
- Indicate if masking was used during group allocation, data collection and/or data analysis

Please outline where this information can be found within the submission (e.g., sections or figure legends), or explain why this information doesn’t apply to your submission:

The 6 most abundant immune cell subtypes were sorted for downstream analysis to meet our mitochondrial phenotyping platform requirements of 5 million cells per sample. Various cell subtypes and participants were interspersed among experimental plates (96-well format) for mitochondrial phenotyping, including samples from the n=21 cohort and n=9 repeated measures, to ensure that all measures across study components (between cell subtypes, between-person, and cohort vs repeat participant) are comparable. This information is summarized in the “Methods” section (page 33).

**Additional data files (“source data”)**

- We encourage you to upload relevant additional data files, such as numerical data that are represented as a graph in a figure, or as a summary table
- Where provided, these should be in the most useful format, and they can be uploaded as “Source data” files linked to a main figure or table
- Include model definition files including the full list of parameters used
- Include code used for data analysis (e.g., R, MatLab)
- Avoid stating that data files are “available upon request”

Please indicate the figures or tables for which source data files have been provided:

All data generated and analyzed during this study, including mitochondrial biochemistry, mtDNA content, and blood chemistry, cell counts from CBC and flow cytometry, and de-identified participant information are included in the supporting data files. Source data files have been provided for Figures 1-9, and for figure supplements (Figure 1-figure supplement 3, Figure 2-figure supplement 1, Figure 4-figure supplement 1, Figure 6-figure supplement 1, Figure 6-figure supplement 2, Figure 6-figure supplement 3, Supplementary file 3, and Appendix 2-figure 1). Requests for resources or other information should be directed to and will be fulfilled by the corresponding author.
